# BMPER Promotes Epithelial-Mesenchymal Transition in the Developing Cardiac Cushions

**DOI:** 10.1371/journal.pone.0139209

**Published:** 2015-09-29

**Authors:** Laura Dyer, Pamela Lockyer, Yaxu Wu, Arnab Saha, Chelsea Cyr, Martin Moser, Xinchun Pi, Cam Patterson

**Affiliations:** 1 McAllister Heart Institute, University of North Carolina at Chapel Hill, Chapel Hill, North Carolina, 27599, United States of America; 2 Department of Pathology and Laboratory Medicine, University of North Carolina at Chapel Hill, Chapel Hill, North Carolina, 27599, United States of America; 3 Cardiology and Angiology I, Heart Center Freiburg University, Freiburg, D-79106, Germany; 4 Cardiovascular Research Institute, Department of Medicine, Athero & Lipo, Baylor College of Medicine, Houston, Texas, 77030, United States of America; 5 New York-Presbyterian Hospital, New York City, New York, 10065, United States of America; Heart Science Centre, Imperial College London, UNITED KINGDOM

## Abstract

Formation of the cardiac valves is an essential component of cardiovascular development. Consistent with the role of the bone morphogenetic protein (BMP) signaling pathway in cardiac valve formation, embryos that are deficient for the BMP regulator BMPER (BMP-binding endothelial regulator) display the cardiac valve anomaly mitral valve prolapse. However, how BMPER deficiency leads to this defect is unknown. Based on its expression pattern in the developing cardiac cushions, we hypothesized that BMPER regulates BMP2-mediated signaling, leading to fine-tuned epithelial-mesenchymal transition (EMT) and extracellular matrix deposition. In the BMPER^-/-^ embryo, EMT is dysregulated in the atrioventricular and outflow tract cushions compared with their wild-type counterparts, as indicated by a significant increase of Sox9-positive cells during cushion formation. However, proliferation is not impaired in the developing BMPER^-/-^ valves. *In vitro* data show that BMPER directly binds BMP2. In cultured endothelial cells, BMPER blocks BMP2-induced Smad activation in a dose-dependent manner. In addition, BMP2 increases the Sox9 protein level, and this increase is inhibited by co-treatment with BMPER. Consistently, in the BMPER^-/-^ embryos, semi-quantitative analysis of Smad activation shows that the canonical BMP pathway is significantly more active in the atrioventricular cushions during EMT. These results indicate that BMPER negatively regulates BMP-induced Smad and Sox9 activity during valve development. Together, these results identify BMPER as a regulator of BMP2-induced cardiac valve development and will contribute to our understanding of valvular defects.

## Introduction

The cardiac valves promote unidirectional flow through the heart, and valve defects are among the most common congenital heart defects [1]. The valves form from an initial swelling in the outflow tract and the atrioventricular canal [2]. The overlying myocardium induces epithelial-mesenchymal transition (EMT) in the underlying endothelial layer, and a subset of these endothelial cells undergoes EMT to populate space between the layers to form cushions. This mesenchymal population is highly proliferative and fills the space between the myocardium and endocardium. As the atrioventricular cushions are populated, they give rise to the mitral and tricuspid valves; in contrast, the outflow tract cushions, which give rise to the semilunar valves, are also populated by migrating cardiac neural crest-derived cells. Once populated, these cushions remodel and elongate, and the extracellular matrix is remodeled; these steps transform the cushions into the mature valves.

One of the earliest signals involved in cushion formation is bone morphogenetic protein (BMP) 2. BMP2 is secreted from the myocardium, and the endocardium expresses BMP receptor 1A [3]. In the developing cushions, BMP2 is sufficient to induce EMT [4] and induces the transcription factors Snail [5], and Twist1 and Msx1/2 [6]. Additionally, BMP2 also induces the transcription factor Sox9 in another population, the neural crest, that also undergoes EMT [7]. All of these transcription factors are required for EMT, and many of them are associated with valve malformations [3]. In addition to regulating EMT in the early stages of cushion formation, Sox9 also promotes elongation and maturation of the mitral and tricuspid valves [8]; however, whether BMP2 promotes this later Sox9 expression is unknown.

Despite the importance of BMP2 in the early stages of cardiac valve development, only intracellular BMP repressor Smad6 has yet been correlated with cardiac valve abnormalities in the Online Mendelian Inheritance in Man database. This discrepancy is highly surprising due to how common these anomalies are and suggests that the BMP pathway is tightly regulated during cardiac development. The BMP pathway can be regulated in numerous ways, including through extracellular proteins. However, the role of BMP regulators in cardiac valve development is understudied. The BMP antagonist noggin can block EMT in excised atrioventricular cushions [9], and noggin is expressed in the myocardium surrounding the developing cushions at E9.5 [10]. Correspondingly, the noggin knockout mouse displays hyperplastic atrioventricular and outflow tract cushions [10]. In the chick, BMP inhibitor follistatin is expressed in the endocardium overlying the developing cardiac cushions [11], but its specific role is unknown.

Previous work in the Patterson laboratory identified a cardiac valve anomaly similar to mitral valve prolapse in mice that are homozygous null for the BMP regulator BMPER (BMP-binding endothelial regulator) [12]. Further, BMPER is expressed in the cardiac valve mesenchyme at E13.5-E14.5 [13], and BMPER has been indirectly shown to interact with BMP2 [14]. Thus, we hypothesized that BMPER modulates BMP2-dependent cardiac valve development. In this study, we show that BMPER is expressed in the developing cardiac cushions and specifically inhibits the canonical BMP intracellular pathway. Further, BMP2-induced EMT is dysregulated in BMPER^-/-^ embryos in a stage-dependent manner, highlighting how tightly the BMP pathway is regulated during EMT. Together, these results support the importance of BMP regulation during cardiac valve development.

## Materials and Methods

### Mice

The BMPER^-/-^ mouse was created previously [15] and is maintained on a mixed C57Bl6/129 background. Because the BMPER^-/-^ embryo is non-viable, embryos were obtained through BMPER^+/-^ timed pregnancies. All experiments were approved by the Institutional Animal Care and Use Committee at the University of North Carolina at Chapel Hill and Baylor College of Medicine.

### Antibodies

The following antibodies were used for immunohistochemistry, co-immunoprecipitation, or Western blot analysis: MF-20 (Cat. No. MF-20; Developmental Studies Hybridoma Bank, Iowa City, IA); PECAM (Cat. No. 553370) and Nfat-c1 (Cat. No. 553370; Becton-Dickinson, San Jose, CA); Sox9 (Cat. No. sc-20095; Santa Cruz Biotechnology, Santa Cruz, CA); Smad1 (Cat. No. 9743S) and phospho-Smad1,5,8 (pSMAD, Cat. No. 13820S; Cell Signaling Technology, Danvers, MA); anti-Crossveinless-2 (referred to as anti-BMPER, Cat. No. AF2299; R&D Systems, Minneapolis, MN); anti-BMP2 (Cat. No. ab14933; Abcam, Cambridge, MA); phalloidin-Texas Red (Cat. No. T7471; Molecular Probes, Grand Island, NY); and anti-ß-actin (Cat. No. A2228; Sigma, St. Louis, MO).

### Histology and immunohistochemistry

For immunohistochemical analyses, embryos were harvested in phosphate-buffered saline (PBS) or 0.1 M Tris buffer (pH 7.6), fixed in 4% formaldehyde overnight at 4°C, washed in PBS or 0.1 M Tris buffer, and processed for frozen sectioning. Unless indicated, immunohistochemistry was performed as described in Waldo et al. [16]. Microwave antigen retrieval using 10 mM citrate buffer (pH 6.0) was required for Nfat-c1, Sox9, and pSMAD. pSMAD immunohistochemistry and semi-quantitative analysis was performed as described in Dyer et al. [13,17]. We processed each litter as a batch to ensure similar conditions throughout the experiment. Within each batch of slides, the slides were examined using identical photography and imaging conditions, and the relative fluorescence intensity in each batch was calculated with respect to the average fluorescence intensity of the littermate wild-types. BMPER immunohistochemistry was performed as described in Zakin et al. [18]. DAPI (Invitrogen, Grand Island, NY) was used as a nuclear counterstain. Slides were examined and imaged as described in Dyer et al. [13].

### BMPER-BMP2 co-immunoprecipitation

Co-immunoprecipitations were performed using recombinant BMPER and BMP2 (both from R&D Systems) as previously described [14]. In brief, beads were conjugated with antibodies recognizing BMPER, BMP2, or the respective IgG controls (goat and rabbit). After incubating antibody-conjugated beads with different combinations of recombinant protein, the beads were isolated and prepared for Western blot.

### BMP activity assay

To determine how BMPER modulated BMP2 activity, mouse cardiac-derived endothelial cells (MECs) were serum starved for 24 h and treated with or without 0.6 nM recombinant BMP2 and increasing doses of recombinant BMPER (0–9.6 nM). Cell lysates were collected after 30 min in Tris-sodium chloride-Tween 20 buffer and analyzed as described in Kelley et al. [15]. All treatments were performed in quadruplicate.

### Western blot analysis

Proteins samples were separated on 4–12% bis-Tris gels and transferred to PVDF membranes; the resulting membranes were blotted for the indicated proteins. For the BMP activity assay, total Smad and β-actin were used as loading controls. Band density was calculated using ImageJ software (www.nih.gov), was normalized to the loading controls, and is presented as the fold-change compared with the untreated samples.

### Statistical analysis

Quantitative data were compared using unpaired Student’s t-test with equal variances in Excel, one or two-way ANOVA and multiple comparisons in Prism. A p-value <0.05 was considered statistically significant.

## Results

### BMPER is expressed throughout the developing cushions

**Fig 1:**
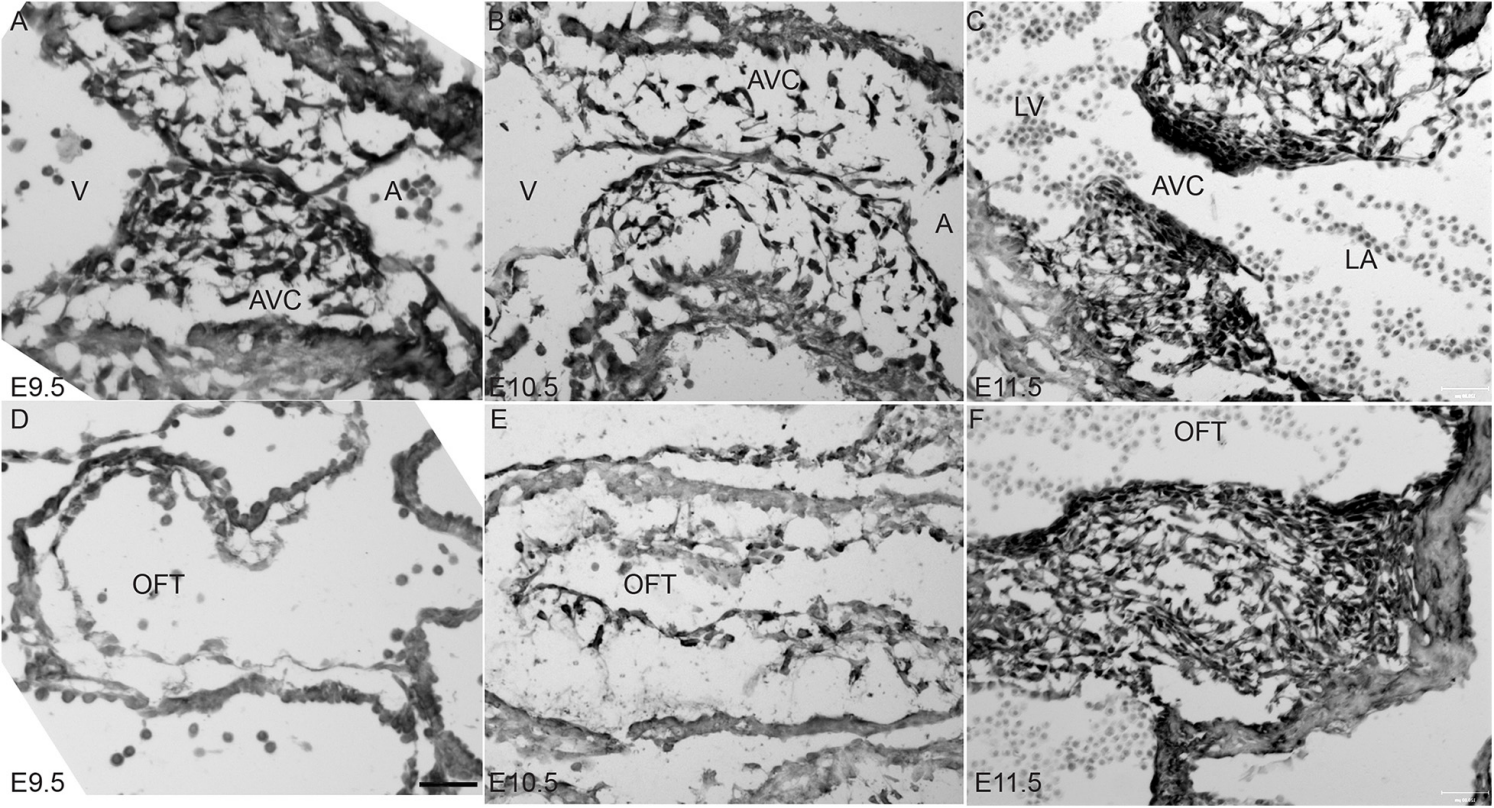
BMPER is expressed in the developing cardiac cushions. BMPER protein expression was evaluated in the atrioventricular cushions (AVC; A-C) and outflow tract cushions (OFT; D-F) at E9.5 (A, D), E10.5 (B, E), and E11.5 (C, F). (A-C) BMPER protein is strongly expressed in the mesenchymal cells that populate the AVCs and is expressed at very low levels in the overlying endocardial layer. (D-F) Consistent with epithelial-mesenchymal transition (EMT) occurring slightly later in the OFT cushions compared with the AVCs (A-C), no BMPER-positive cells are observed in the OFT at E9.5 (D). As EMT progresses (E-F), the OFT cushions fill with BMPER-positive mesenchymal cells. Scale bar in D = 60 μm in panels A, B, D, and E and = 120 μm in panels C and F. A, common atrium; V, common ventricle; LA, left atrium; LV, left ventricle.

Because the BMP pathway promotes normal valve development [3] and the BMPER^-/-^ embryo presents with mitral valve anomalies at E18.5 [12], we wanted to assess whether BMPER affects the early stages of valve formation. Thus, BMPER protein expression was examined between E9.5 and E11.5. At E9.5, the mesenchymal cells populating the atrioventricular cushions have begun expressing BMPER, whereas no expression is present yet in the outflow tract cushions (Fig 1A and 1D). By E10.5, the mesenchymal cells that populate both the atrioventricular and outflow tract cushions express BMPER (Fig 1B and 1E), and expression is maintained through E11.5 in both sets of cushions (Fig 1C and 1F). As expected, no BMPER protein is detected in BMPER^-/-^ embryos (data not shown and [13]). Interestingly, BMPER expression in the overlying endothelial layer appears much weaker with respect to the mesenchyme. BMPER remains expressed in the mesenchymal cells through at least E14.5 [12]. BMPER’s more robust expression within the mesenchyme suggests that it does not affect the initiation of EMT but may have later effects during EMT.

### EMT is dysregulated in BMPER^-/-^ embryos

**Fig 2:**
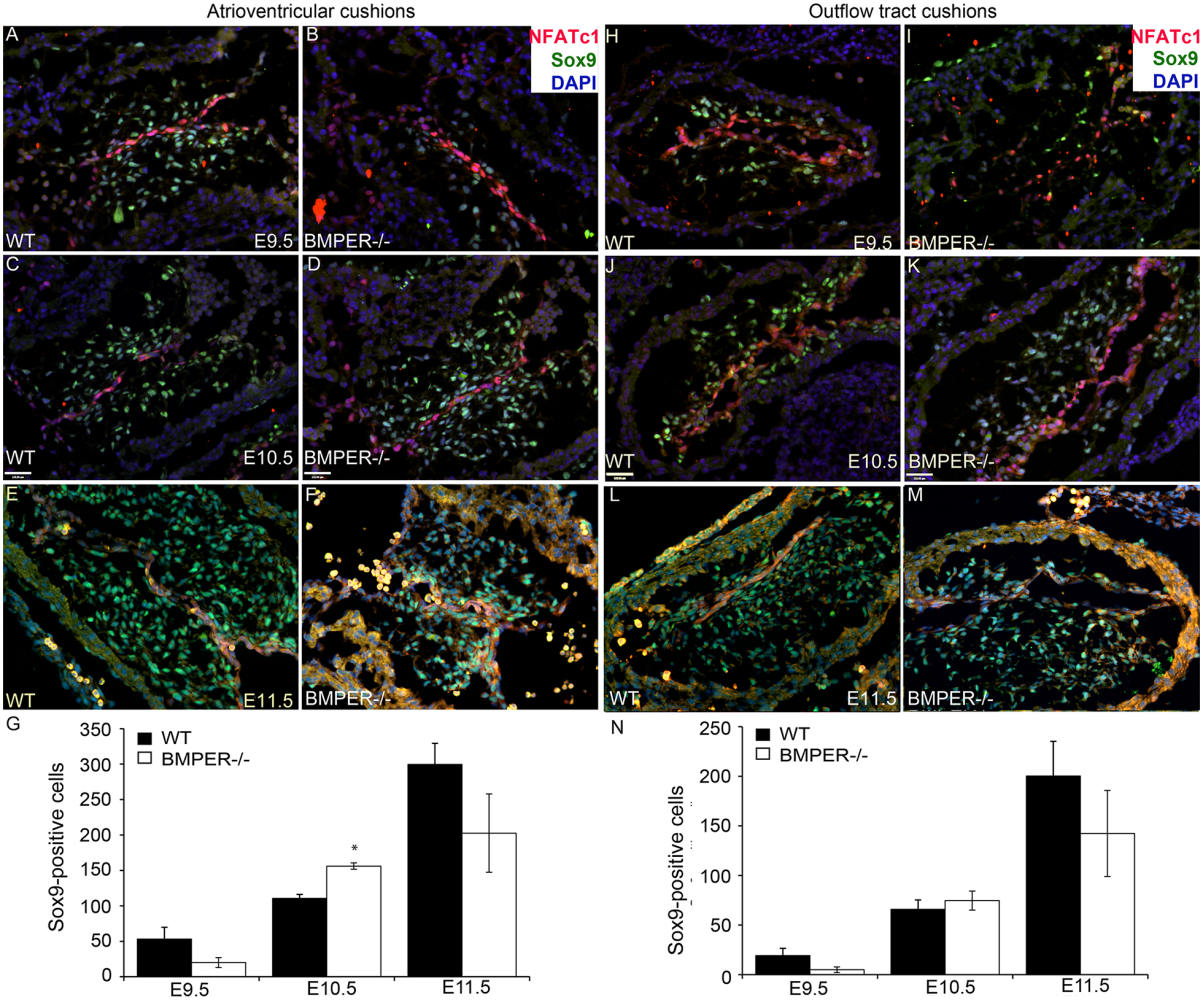
EMT is dysregulated in the BMPER^-/-^ cushions. EMT markers Nfat-c1 (red) and Sox9 (green) were assessed in the atrioventricular cushions (AVCs; A-G) and outflow tract (OFT) cushions (H-N) at E9.5-E11.5. In wild-type (A, C, E) and BMPER^-/-^ (B, D, F) AVCs at E9.5 (A, B), E10.5 (C, D), and E11.5 (E, F), Nfat-c1 expression appears identical between genotypes at each time point. In contrast, BMPER^-/-^ embryos tend to have fewer Sox9-positive cells at E9.5 (B; p<0.1, quantified in G). By E10.5, the number of Sox9-positive cells is significantly higher in the BMPER^-/-^ AVCs (D; *, p<0.01) compared with the wild-type AVCs (C). This pattern is not maintained, with a non-significant reduction in Sox9-positive cells present in the BMPER^-/-^ AVCs by E11.5 (compare F with wild-type in E). (G) The number of Sox9-positive cells was counted in 2–4 sagittal sections, and the two most populated AVCs per embryo were averaged and used to compare how the cushions were populated. n = 6, 5, and 4 WT AVCs (black bars) and 6, 7, and 4 BMPER^-/-^ AVCs (white bars) at E9.5, E10.5, and E11.5, respectively. (H-N) Nfat-c1 expression in the OFT cushions appears identical between genotypes at each time point. Though not statistically significant, BMPER^-/-^ OFT cushions show the same pattern of Sox9-positive cell counts as the AVCs, with a tendency toward fewer Sox9-positive cells at E9.5 (I compared with WT in H, quantified in N), more Sox9-positive cells at E10.5 (K compared with WT in J), and fewer Sox9-positive cells at E11.5 (M compared with WT in L). (N) The number of Sox9-positive cells was counted as describe in (G). n = 6, 7, and 4 WT OFT cushions (black bars) and 6, 8, and 4 BMPER^-/-^ OFT cushions (white bars) at E9.5, E10.5, and E11.5, respectively. Student’s t-test was used to compare means between genotypes. Scale bars in C and D = 120 μm and apply to A-F and H-M.

At E18.5, the BMPER^-/-^ embryo displays a mitral valve phenotype that is consistent with mitral valve prolapse [12]. Thus, in combination with the increased expression of BMPER in the developing cushions when EMT takes place and BMP2’s critical role in EMT [4], we hypothesized that BMPER would affect EMT. We evaluated the expression of two EMT markers: Nfat-c1, which is an early marker of EMT [20], and Sox9, which is a late marker of EMT and a known BMP2 target [7]. In the atrioventricular cushions, Nfat-c1 expression was observed in the overlying endocardium at E9.5–11.5 in both the wild-type and BMPER^-/-^ embryos, with no apparent differences (Fig 2A–2F, red signal). In contrast, non-significantly fewer Sox9-positive cells were initially observed in the BMPER^-/-^ atrioventricular cushions at E9.5 (Fig 2B, compared with 2A, green signal, and 2G). However, by E10.5, significantly more Sox9-positive cells were present in the BMPER^-/-^ atrioventricular cushions (Fig 2D, green signal, and 2G). Interestingly, although the total number of Sox9-positive cells increased at E10.5, the percentage of mesenchymal cells that expressed Sox9 non-significantly decreased at E10.5 (82.5% in the BMPER^-/-^ vs. 86.8% in the wild-type atrioventricular cushions), indicating that more Sox9-negative mesenchymal cells were also present in the BMPER^-/-^ atrioventricular cushions. The increase in total Sox9-positive cells was short-lived; between E10.5 and E11.5, the number of wild-type Sox9-positive cells increased greatly, whereas the BMPER^-/-^ Sox9-positive cells showed only a slight increase, leading to fewer overall Sox9-positive mesenchymal cells in the BMPER^-/-^ atrioventricular cushions compared with the wild-type cushions (Fig 2E and 2F, green signal and 2G). Additionally, the percentage of mesenchymal cells that were Sox9-positive was also significantly decreased (72.2% in the BMPER^-/-^ vs. 92.3% in the wild-type cushions, p<0.01), suggesting that a subset of mesenchymal cells have prematurely stopped expressing Sox9. A similar, though non-significant, pattern was observed in the outflow tract cushions (Fig 2H–2N). The disruption of Sox9 expression, but not Nfat-c1 expression, is consistent with BMPER’s expression within the forming mesenchymal cells but exclusion from the overlying endocardium. Together, these data suggest that EMT is dysregulated in the absence of BMPER but that a secondary mechanism quickly compensates for the lack of BMPER.

### Mesenchymal proliferation is not regulated by BMPER

**Fig 3:**
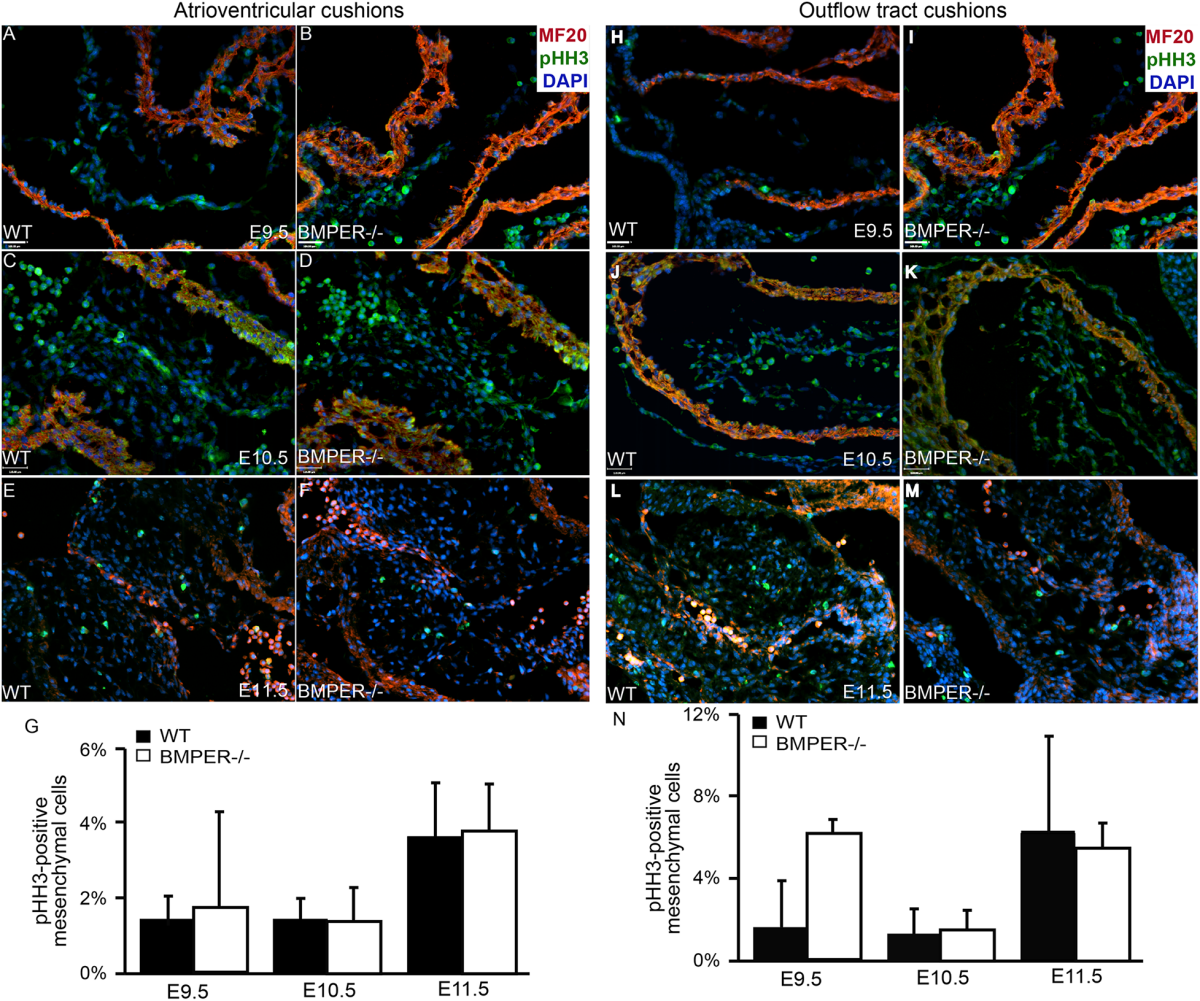
Proliferation is normal in the BMPER^-/-^ cushions. Proliferation was assessed in the atrioventricular cushions (AVCs) and outflow tract (OFT) cushions. Proliferative cells were detected via phosphohistone H3 expression (green), and sagittal sections were colabeled with the myocardial marker MF20 (red) and nuclear marker DAPI (blue). For each sample, all mesenchymal cells in at least 3 sections or a minimum of 100 cells were counted. (A-G) Wild-type (A, C, E) and BMPER^-/-^ (B, D, F) AVCs were evaluated at E9.5 (A, B), E10.5 (C, D), and E11.5 (E, F). (A, B) At E9.5, no significant differences were observed between genotypes. (C, D) By E10.5, the proliferation remained similar in both the BMPER^-/-^ and wild-type AVCs. (E, F) By E11.5, the proliferation rate increased similarly in both genotypes. (G) The proliferation rates for each group were quantified. n = 2, 4, and 3 for wild-type AVCs and 2, 5, and 3 for BMPER-/- AVCs at E9.5, E10.5, and E11.5, respectively. (H-N) Wild-type and BMPER^-/-^ OFT cushions were evaluated in the same manner. (H, I) At E9.5, proliferation was increased, though not significantly, in the OFT cushions of BMPER^-/-^ embryos compared with wild-type embryos. (J, K) By E10.5, the proliferation rate decreased in the BMPER^-/-^ OFT cushions and was comparable to that in the wild-type OFT cushions. (L, M) As EMT ended and the OFT cushions entered the proliferative phase, the proliferation rate increased similarly in both genotypes. (N) The proliferation rates for each group were quantified. n = 2, 4, and 3 for wild-type OFT cushions and 2, 5, and 3 for BMPER^-/-^ OFT cushions at E9.5, E10.5, and E11.5, respectively. Scale bars in A, B, H, and I = 100 μm; scale bars in C, D, J, and K = 110 μm and apply to E, F, L, and M.

As a potential mechanism to explain the sudden increase in Sox9-positive cells in the BMPER^-/-^ atrioventricular cushions at E10.5, we examined proliferation at E9.5-E11.5. Surprisingly, though, no differences were observed in the proliferation rates in the BMPER^-/-^ atrioventricular cushions compared with their wild-type counterparts at any time point examined (Fig 3A–3G). Additionally, no statistically significant differences in the proliferation rate were observed between genotypes in the outflow tract cushions at any time point examined; however, there was a non-significant increase at E9.5 in the BMPER^-/-^ outflow tract cushions (Fig 3H–3N). These results suggest that the increase in Sox9-positive cell number at E10.5 is not due to an increase in cell proliferation. Other unknown mechanisms involved in this process remain to be determined.

### BMPER interacts with BMP2 and modulates BMP2-mediated signaling

**Fig 4:**
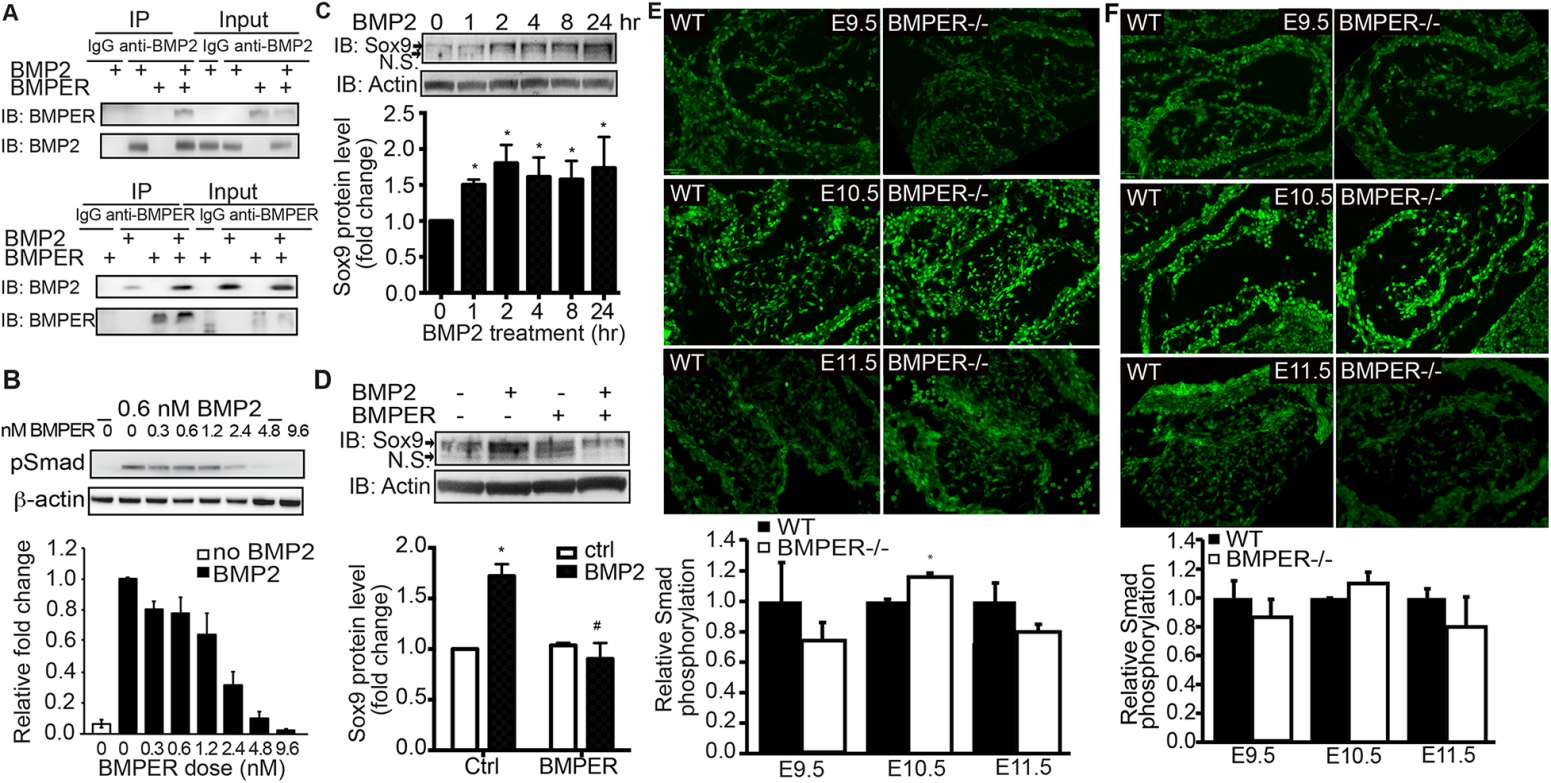
BMPER inhibits BMP2-induced signaling in the developing cushions. (A) Recombinant BMPER and BMP2 proteins were combined as indicated and immunoprecipitated using an anti-BMPER antibody, an anti-BMP2 antibody, or the appropriate species-specific IgG antibody controls. As a loading control, all unbound proteins in the supernatant were run in the Input lanes (right lanes). The anti-BMP2 antibody recognized recombinant BMP2 and co-immunoprecipitated BMPER when both proteins were present. Similarly, the anti-BMPER antibody recognized recombinant BMPER and co-immunoprecipitated BMP2 when both proteins were present. (B) BMPER inhibits BMP2-induced Smad1,5,8 phosphorylation (pSmad) in cultured endothelial cells. MECs were treated with BMP2 and increasing doses of BMPER for 45 minutes. As expected, BMP2 treatment induced Smad phosphorylation (second lane). With increasing concentrations of BMPER, the pSmad levels were exponentially reduced (R^2^ = 0.98). Data are presented as the fold change compared with BMP2 treatment in the absence of BMPER treatment. Due to reduced signaling intensity when stripping and reprobing blots, β-actin was used as a loading control instead of total Smad. (C) BMP2 increases the Sox9 protein level in cultured endothelial cells. MECs were treated with 0.6 nM BMP2 for the indicated time periods. As expected, BMP2 treatment increased Sox9 protein levels. The arrow indicates the Sox9 protein band. N.S., not significant. *p<0.05, compared with cells without treatment. n = 3. (D) BMPER inhibits BMP2-induced Sox9 protein expression in cultured endothelial cells. MECs were treated with 0.6 nM BMP2 and 5 nM BMPER for 4 hours. BMPER co-treatment blocks BMP2-induced Sox9 protein expression. The arrow indicates the Sox9 protein band. N.S., not significant. *p<0.05, compared with control cells without treatment; #p<0.05, compared with cells with BMP2 treatment only. n = 3. (E) BMPER affects downstream Smad1/5/8 activity in the developing atrioventricular cushions. At E9.5, BMPER^-/-^ atrioventricular cushions display reduced pSmad signals compared with their wild-type counterparts. However, by E10.5, the pSmad intensity increases in the BMPER^-/-^ atrioventricular cushions compared with the wild-type counterparts. This increase is not maintained, with reduced pSmad intensity in the BMPER^-/-^ cushions by E11.5. Fluorescence intensity is quantified on the right. (F) At E9.5, BMPER^-/-^ outflow tract cushions display reduced pSmad compared with their wild-type counterparts. However, by E10.5, the pSmad intensity increases in the BMPER^-/-^ outflow tract cushions compared with the wild-type counterparts. This increase is not maintained, with reduced pSmad intensity in the BMPER^-/-^ cushions by E11.5. *p<0.05. Scale bar = 120 μm.

Previous work has directly shown that BMPER interacts with BMP4 and indirectly shown that BMPER also interacts with BMP2 [14]. Because BMP2 is the major BMP involved in cardiac cushion development by inducing Sox9 [6], we tested the direct interaction of BMPER-BMP2 using co-immunoprecipitation with recombinant proteins BMPER and BMP2. As a positive control, the BMP2 antibody and the BMPER antibody both successfully immunoprecipitated BMP2 and BMPER, respectively (Fig 4A). In the presence of both proteins, both antibodies could immunoprecipitate both proteins (Fig 4A), thus indicating that BMPER and BMP2 directly interact *in vitro*.

Because BMPER regulates BMP4-induced activity [15] and BMPER and BMP2 interact *in vitro*, we wanted to determine what the effect of BMPER was on BMP2-induced signaling and Sox9 induction. Ideally, these effects would have been tested using *ex vivo* cultured cushions or embryonic mesenchymal cells. Due to technical difficulties with the *ex vivo* collagen gel technique and to allow for more similar comparisons with previous BMPER research [15,19], MECs were utilized for these experiments. MECs were treated with a fixed concentration of BMP2 and increasing concentrations of BMPER. Unlike BMPER’s ability to both promote and inhibit BMP4, BMPER serves only as an inhibitor of BMP2-induced Smad phosphorylation (Fig 4B). In response to BMP2 treatment alone, Smad phosphorylation increases quickly in MECs, and this response is inhibited exponentially by increasing concentrations of BMPER. In addition, BMP2 treatment increases Sox9 protein expression as early as one hour after stimulation (Fig 4C), consistent with the published data [7]. More interestingly, BMPER co-treatment significantly blocks Sox9 protein expression induced by BMP2 (Fig 4D). All these data indicate that BMPER negatively regulates BMP2-induced Smad activity and Sox9 protein induction in cultured MECs.

Because BMPER blocks BMP2 activity in cultured MECs, we hypothesized that Smad activation would increase *in vivo* in BMPER^-/-^ embryos. When examining Smad phosphorylation levels in BMPER^-/-^ embryos with immunofluorescence using a relative fluorescence intensity as an indicator of signaling activity, the pSmad intensity is non-significantly lower in the mesenchymal cells populating atrioventricular and outflow tract cushions compared with their wild-type counterparts at E9.5 (Fig 4E and 4F). Additionally, no differences were observed between genotypes in the outflow tract myocardium, which does not express BMPER (data not shown). However, this BMP activity pattern changes at E10.5, with significantly more intense pSmad signals in the BMPER^-/-^ cushions, as expected based on the *in vitro* data. By E11.5, pSmad expression remains slightly reduced in the atrioventricular cushions of the BMPER^-/-^ embryos, and no changes are observed in the outflow tract cushions (Fig 4E and 4F). In combination with the *in vitro* data in MECs, these data suggest that BMPER regulates EMT process by fine-tuning BMP activity at specific time points in these developing cushions. However, additional regulatory components are likely also involved during valve development.

## Discussion

The BMP pathway, particularly BMP2, is the major driving factor behind cardiac valve formation. The knockout of either BMP2 in the atrioventricular myocardium or receptor BMPR1 in the endocardium leads to, respectively, a complete or nearly complete absence of cushion development by blocking EMT [6,21–23]. Here, we show that the BMP regulator BMPER inhibits BMP2-dependent signaling and fine-tunes normal valve development. In the absence of BMPER, EMT in the cardiac cushions, particularly the atrioventricular cushions, is dysregulated. We hypothesize that this dysregulation underlies the mitral valve prolapse observed in E18.5 BMPER^-/-^ embryos [12]. A similar valve phenotype is observed when BMP receptor II is conditionally knocked out of the endocardium [24], and knockout of the BMP repressor Smad6 leads to hyperplasia of the developing cushions [25]. These reports and our data support BMPER’s requisite role in this specific context.

The number of Sox9-positive cells in the BMPER^-/-^ atrioventricular cushions increased dramatically between E9.5 and E10.5, compared with the more modest increase observed in the wild-type atrioventricular cushions. These cell number data are particularly intriguing because no increase in proliferation is observed in the BMPER^-/-^ atrioventricular cushions at these embryonic stages. Thus, how does the BMPER^-/-^ embryo generate more Sox9-positive cells than the wild-type embryo? Epicardial cells contribute to the parietal leaflets of the atrioventricular valves through epithelial-mesenchymal transition, but this addition occurs at later stages than those observed herein [26]. In the outflow tract, migrating cardiac neural crest-derived cells [27] could explain these differences, but the BMPER^-/-^ outflow tract cushions also do not show differences in cell number. Neither of these populations thus appears to be a viable mechanism, and the mechanism through which the BMPER^-/-^ embryo recovers the number of Sox9-positive atrioventricular cells remains elusive. One other possibility is related to the lower proportion of mesenchymal cells that express Sox9 in the BMPER^-/-^ atrioventricular cushions at E11.5. Perhaps this increase in Sox9-negative cells drives the initiation of EMT in additional epithelial cells, or perhaps some of the increased Sox9-positive cells observed at E10.5 prematurely turn off Sox9. Alternatively, BMP2 promotes the expression of repressor Smad6 to restrict EMT [6]; thus, the absence of BMPER may lead to the up-regulation of Smad6 via BMP2, halting EMT and leading to the reduced percentage of Sox9-positive mesenchymal cells over time.

This ability to so carefully regulate cell number is dramatic and demonstrates the plasticity of the early embryo. In similarly aged chicken embryos, previous work has shown that the elevation of one signaling pathway can lead to compensatory upregulation in a regulatory pathway within as little as 12 hours [17]. While in this prior study, the BMP pathway reduced proliferation by regulating the Sonic hedgehog pathway, these results highlight the quickness with which cardiac progenitors can respond to aberrant signaling. A similar regulatory event may balance the elevated BMP signaling in the developing BMPER^-/-^ valves.

Despite the temporal changes observed in the BMPER^-/-^ atrioventricular cushions, our results are consistent with BMPER playing a negative regulatory role in the BMP pathway in this context. Knocking out the BMP antagonist noggin results in the opposite phenotype, with excess EMT, overproliferation, and hyperplastic cardiac cushions [10], and knockout of the repressor Smad6 similarly shows hyperplastic cardiac cushions [25]. The reduction of BMP4 signaling that occurs in a conditional knockout mouse lacking FGF receptors 1 and 2 in the heart field progenitors is also accompanied by hypertrophic semilunar valves [28]. Our BMPER deletion increased the Sox9-positive cell number at E10.5, suggesting hyperplasia (Fig 2). However, this increase is temporary and is not due to overproliferation. Additionally, the number of Sox9-negative cells also increases by E11.5 in the atrioventricular cushions of the BMPER^-/-^ embryos. Together, these results suggest that additional mechanisms exist to compensate BMPER activity and regulate EMT. Excess mesenchymal cells are also observed in the Smad6 knockout mouse [25], supporting the role of the BMP pathway in capping the number of cells that populate the cushions. These results suggest that only a narrow range of BMP signaling can yield normal valves during development and highlight the necessity of understanding how the BMP pathway is so carefully regulated. Further experimentation is required to identify other components of the BMP regulatory complex and determine whether these regulators are associated with human congenital valve anomalies.

## References

[1] Roger VL, Go AS, Lloyd-Jones DM, Benjamin EJ, Berry JD, Borden WB, et al (2012) Heart disease and stroke statistics-- update: A report from the american heart association. Circulation 125: e2-e220. 10.1161/CIR.0b013e31823ac046 22179539 PMC4440543

[2] Hinton RB, Yutzey KE (2011) Heart valve structure and function in development and disease. Annu Rev Physiol 73: 29–46. 10.1146/annurev-physiol-012110-142145 20809794 PMC4209403

[3] Chakraborty S, Combs MD, Yutzey KE (2010) Transcriptional regulation of heart valve progenitor cells. Pediatric cardiology 31: 414–421. 10.1007/s00246-009-9616-x 20039031 PMC2837124

[4] Runyan RB, Markwald RR (1983) Invasion of mesenchyme into three-dimensional collagen gels: A regional and temporal analysis of interaction in embryonic heart tissue. Dev Biol 95: 108–114. 6825921 10.1016/0012-1606(83)90010-6

[5] Luna-Zurita L, Prados B, Grego-Bessa J, Luxan G, del Monte G, Benguria A, et al (2010) Integration of a notch-dependent mesenchymal gene program and bmp2-driven cell invasiveness regulates murine cardiac valve formation. The Journal of clinical investigation 120: 3493–3507. 10.1172/JCI42666 20890042 PMC2947227

[6] Ma L, Lu MF, Schwartz RJ, Martin JF (2005) Bmp2 is essential for cardiac cushion epithelial-mesenchymal transition and myocardial patterning. Development 132: 5601–5611. 16314491 10.1242/dev.02156

[7] Sakai D, Suzuki T, Osumi N, Wakamatsu Y (2006) Cooperative action of sox9, snail2 and pka signaling in early neural crest development. Development 133: 1323–1333. 16510505 10.1242/dev.02297

[8] Lincoln J, Kist R, Scherer G, Yutzey KE (2007) Sox9 is required for precursor cell expansion and extracellular matrix organization during mouse heart valve development. Developmental Biology 305: 120–132. 17350610 10.1016/j.ydbio.2007.02.002 PMC1920559

[9] Sugi Y, Yamamura H, Okagawa H, Markwald RR (2004) Bone morphogenetic protein-2 can mediate myocardial regulation of atrioventricular cushion mesenchymal cell formation in mice. Dev Biol 269: 505–518. 15110716 10.1016/j.ydbio.2004.01.045

[10] Choi M, Stottmann RW, Yang YP, Meyers EN, Klingensmith J (2007) The bone morphogenetic protein antagonist noggin regulates mammalian cardiac morphogenesis. Circ Res 100: 220–228. 17218603 10.1161/01.RES.0000257780.60484.6a

[11] van den Berg G, Somi S, Buffing AA, Moorman AF, van den Hoff MJ (2007) Patterns of expression of the follistatin and follistatin-like1 genes during chicken heart development: A potential role in valvulogenesis and late heart muscle cell formation. Anat Rec (Hoboken) 290: 783–787. 17549728 10.1002/ar.20559

[12] Willis MS, Dyer LA, Ren R, Lockyer P, Moreno-Miralles I, Schisler JC, et al (2012) Bmper regulates cardiomyocyte size and vessel density in vivo. Cardiovascular pathology: the official journal of the Society for Cardiovascular Pathology: 228–240. 23200275 10.1016/j.carpath.2012.10.005 PMC5027882

[13] Dyer LA, Wu Y, Moser M, Patterson C (2014) Bmper-induced bmp signaling promotes coronary artery remodeling. Developmental Biology 386: 385–394. 10.1016/j.ydbio.2013.12.019 24373957 PMC4112092

[14] Moser M, Binder O, Wu Y, Aitsebaomo J, Ren R, Bode C, et al (2003) Bmper, a novel endothelial cell precursor-derived protein, antagonizes bone morphogenetic protein signaling and endothelial cell differentiation. Mol Cell Biol 23: 5664–5679. 12897139 10.1128/MCB.23.16.5664-5679.2003 PMC166349

[15] Kelley R, Ren R, Pi X, Wu Y, Moreno I, Willis M, et al (2009) A concentration-dependent endocytic trap and sink mechanism converts bmper from an activator to an inhibitor of bmp signaling. J Cell Biol 184: 597–609. 10.1083/jcb.200808064 19221194 PMC2654123

[16] Waldo KL, Kumiski D, Kirby ML (1996) Cardiac neural crest is essential for the persistence rather than the formation of an arch artery. Dev Dyn 205: 281–292. 8850564 10.1002/(SICI)1097-0177(199603)205:3<281::AID-AJA8>3.0.CO;2-E

[17] Dyer LA, Makadia FA, Scott A, Pegram K, Hutson MR, Kirby ML, et al (2010) Bmp signaling modulates hedgehog-induced secondary heart field proliferation. Developmental Biology 348: 167–176. 10.1016/j.ydbio.2010.09.021 20920499 PMC2982885

[18] Zakin L, Metzinger CA, Chang EY, Coffinier C, De Robertis EM (2008) Development of the vertebral morphogenetic field in the mouse: Interactions between crossveinless-2 and twisted gastrulation. Dev Biol 323: 6–18. 10.1016/j.ydbio.2008.08.019 18789316 PMC2647368

[19] Moreno-Miralles I, Ren R, Moser M, Hartnett ME, Patterson C (2011) Bone morphogenetic protein endothelial cell precursor-derived regulator regulates retinal angiogenesis in vivo in a mouse model of oxygen-induced retinopathy. Arteriosclerosis, thrombosis, and vascular biology 31: 2216–2222. 10.1161/ATVBAHA.111.230235 21737784 PMC3184390

[20] Chang CP, Neilson JR, Bayle JH, Gestwicki JE, Kuo A, Stankunas K, et al (2004) A field of myocardial-endocardial nfat signaling underlies heart valve morphogenesis. Cell 118: 649–663. 15339668 10.1016/j.cell.2004.08.010

[21] Rivera-Feliciano J, Tabin CJ (2006) Bmp2 instructs cardiac progenitors to form the heart-valve-inducing field. Dev Biol 295: 580–588. 16730346 10.1016/j.ydbio.2006.03.043 PMC2680002

[22] Song L, Fassler R, Mishina Y, Jiao K, Baldwin HS (2007) Essential functions of alk3 during av cushion morphogenesis in mouse embryonic hearts. Dev Biol 301: 276–286. 16959237 10.1016/j.ydbio.2006.08.004

[23] Park C, Lavine K, Mishina Y, Deng CX, Ornitz DM, Choi K (2006) Bone morphogenetic protein receptor 1a signaling is dispensable for hematopoietic development but essential for vessel and atrioventricular endocardial cushion formation. Development 133: 3473–3484. 16887829 10.1242/dev.02499

[24] Beppu H, Ichinose F, Kawai N, Jones RC, Yu PB, Zapol WM, et al (2004) Bmpr-ii heterozygous mice have mild pulmonary hypertension and an impaired pulmonary vascular remodeling response to prolonged hypoxia. Am J Physiol Lung Cell Mol Physiol 287: L1241-1247. 15286002 10.1152/ajplung.00239.2004

[25] Galvin KM, Donovan MJ, Lynch CA, Meyer RI, Paul RJ, Lorenz JN, et al (2000) A role for smad6 in development and homeostasis of the cardiovascular system. Nat Genet 24: 171–174. 10655064 10.1038/72835

[26] Lockhart MM, Boukens BJ, Phelps AL, Brown CL, Toomer KA, Burns TA, et al (2014) Alk3 mediated bmp signaling controls the contribution of epicardially derived cells to the tissues of the atrioventricular junction. Dev Biol 396: 8–18. 10.1016/j.ydbio.2014.09.031 25300579 PMC4252836

[27] Kirby ML, Gale TF, Stewart DE (1983) Neural crest cells contribute to normal aorticopulmonary septation. Science 220: 1059–1061. 6844926 10.1126/science.6844926

[28] Zhang J, Chang JY, Huang Y, Lin X, Luo Y, Schwartz RJ, et al (2010) The fgf-bmp signaling axis regulates outflow tract valve primordium formation by promoting cushion neural crest cell differentiation. Circ Res: 1209–1219. 10.1161/CIRCRESAHA.110.225318 20847311 PMC3052773

