# Correction: BMPER Promotes Epithelial-Mesenchymal Transition in the Developing Cardiac Cushions

**DOI:** 10.1371/journal.pone.0207504

**Published:** 2018-11-29

**Authors:** Laura Dyer, Pamela Lockyer, Yaxu Wu, Arnab Saha, Chelsea Cyr, Martin Moser, Xinchun Pi, Cam Patterson

After publication of the article, concerns were raised regarding Fig 3. Specifically, panel B in "Atrioventricular cushions" is duplicated in panel I in "Outflow tract cushions".

The authors have acknowledged accidental errors in the preparation of this figure. When originally submitting this manuscript, the non-significant outflow tract data were included as supplemental figures. When constructing the original Fig 3 and supplemental Figure 3, LD had identified which samples were most representative for all times and genotypes except for the E9.5 BMPER-/- OFT image, for which no embryo was selected. When identifying which sample would best represent the E9.5 BMPER-/- OFT data, LD accidentally used the notes for the E9.5 BMPER-/- AVC data and duplicated the AVC data. As part of a supplemental image, the duplicated figure went unnoticed until through the review and revision process, even as the supplemental figures were incorporated into the main figures.

The authors are glad for this opportunity to correct panel 3I in Fig 3. Please see the new Fig 3 here.

**Fig 3:**
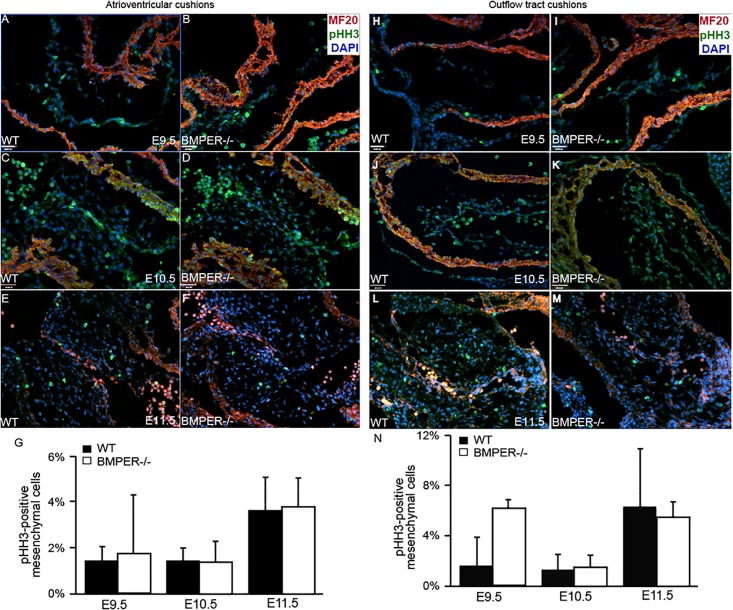
Proliferation is normal in the BMPER^-/-^ cushions. Proliferation was assessed in the atrioventricular cushions (AVCs) and outflow tract (OFT) cushions. Proliferative cells were detected via phosphohistone H3 expression (green), and sagittal sections were colabeled with the myocardial marker MF20 (red) and nuclear marker DAPI (blue). For each sample, all mesenchymal cells in at least 3 sections or a minimum of 100 cells were counted. (A-G) Wild-type (A, C, E) and BMPER^-/-^ (B, D, F) AVCs were evaluated at E9.5 (A, B), E10.5 (C, D), and E11.5 (E, F). (A, B) At E9.5, no significant differences were observed between genotypes. (C, D) By E10.5, the proliferation remained similar in both the BMPER^-/-^ and wild-type AVCs. (E, F) By E11.5, the proliferation rate increased similarly in both genotypes. (G) The proliferation rates for each group were quantified. n = 2, 4, and 3 for wild-type AVCs and 2, 5, and 3 for BMPER^-/-^ AVCs at E9.5, E10.5, and E11.5, respectively. (H-N) Wild-type and BMPER^-/-^ OFT cushions were evaluated in the same manner. (H, I) At E9.5, proliferation was increased, though not significantly, in the OFT cushions of BMPER^-/-^ embryos compared with wild-type embryos. (J, K) By E10.5, the proliferation rate decreased in the BMPER^-/-^ OFT cushions and was comparable to that in the wild-type OFT cushions. (L, M) As EMT ended and the OFT cushions entered the proliferative phase, the proliferation rate increased similarly in both genotypes. (N) The proliferation rates for each group were quantified. n = 2, 4, and 3 for wild-type OFT cushions and 2, 5, and 3 for BMPER^-/-^ OFT cushions at E9.5, E10.5, and E11.5, respectively. Scale bars in A, B, H, and I = 100 μm; scale bars in C, D, J, and K = 110 μm and apply to E, F, L, and M.

The authors have also provided the raw data underlying their figures and results presented in the article. The data can be accessed in the Harvard Dataverse at the following URL: https://dataverse.harvard.edu/dataverse/BMPER_EMT.
